# Online Learning during the COVID-19 Pandemic: Dental Students’ Perspective and Impact on Academic Performance, One Institution Experience

**DOI:** 10.3390/dj10070131

**Published:** 2022-07-11

**Authors:** Widya Lestari, Solachuddin J. A. Ichwan, Siti Zakirah Yaakop, Nurina Sabaznur, Azlini Ismail, Cortino Sukotjo

**Affiliations:** 1 Department of Fundamental Dental and Medical Sciences, Kulliyyah of Dentistry, International Islamic University Malaysia Kuantan Campus, Jalan Sultan Ahmad Shah, Bandar Indera Mahkota, Kuantan 25200, Malaysia; drwidya@iium.edu.my (W.L.); dr_azlini@iium.edu.my (A.I.); 2 Dentistry Programme PAPRSB Institute of Health Sciences, Universiti Brunei Darussalam, Jalan Tungku Link, Gadong BE 1410, Brunei; solachuddin.ichwan@ubd.edu.bn; 3 Kulliyyah of Dentistry, International Islamic University Malaysia Kuantan Campus, Jalan Sultan Ahmad Shah, Bandar Indera Mahkota, Kuantan 25200, Malaysia; siti.zakirah.2412@gmail.com (S.Z.Y.); ninasabaz1@gmail.com (N.S.); 4 Department of Restorative Dentistry, University of Illinois Chicago College of Dentistry, 801 S Paulina St., Chicago, IL 60612, USA

**Keywords:** COVID-19, online learning, students’ perspective, academic performance

## Abstract

Background: The COVID-19 pandemic caused all universities in Malaysia to switch to online learning, including for dental education. The effect of this switch has yet to be assessed. Thus, this study aimed to assess International Islamic University Malaysia (IIUM) dental students’ perspectives on the implementation of online learning during the COVID-19 pandemic and its impact on academic performance. Methods: Cross-sectional and retrospective methods were used. The handling, didactic benefits, motivation, and challenges of online learning were assessed via an online questionnaire, and academic performance was assessed by comparing professional exam scores pre- and post-online learning. Results: Among the 249 IIUM dental student respondents, a positive response was recorded for the management of online learning, despite a few challenges in the area of didactic benefits and motivation. A significant improvement (*p*-value < 0.05) was observed in examination scores in oral biology, microbiology, and pharmacology, while dental material and GMGS showed declines in performance. Other subjects showed no significant difference (*p*-value > 0.05) in mean scores before and after online learning. Conclusion: Generally, students had a positive response towards online learning management, despite facing some challenges. Based on the analysis of examination results, only two subjects in Year 2 and Year 3 were negatively affected by online learning during the pandemic.

## 1. Introduction

The COVID-19 pandemic led to a sudden shutdown of institutions in 2020, including in Malaysia. On 18 March 2020, the government of Malaysia implemented a Movement Control Order to encourage the public to stay at home and practice social distancing [1]. As a consequence of this pandemic, all universities in Malaysia were strongly affected and were forced to face the new challenge of implementing “distance/online learning” [2].

The Kulliyyah of Dentistry (KOD), International Islamic University Malaysia (IIUM) was one of the faculties forced to embrace online learning for the first time. Approximately 90% of the faculty members used a synchronous form of online learning and supplemented this with the asynchronous form. In September 2020, Malaysia entered the third wave of the COVID-19 pandemic. On 2 October 2020, the Ministry of Higher Education officially announced the adjournment of face-to-face registration for admissions in October 2020, resulting in the continuation of distance learning in all universities [3]. This unprecedented changes in learning methods had raised a few concerns, especially regarding students’ satisfaction regarding the online learning system. Some studies conducted showed that students gave a positive response towards online learning [4,5]; however, there were also studies that reported students’ negative experiences [6,7]. A previous study from Justus Liebig University in Germany showed a positive attitude of the students towards online learning, such as it yields higher motivation in learning, easier participation, and less time effort used [8].

In dental education, online learning has vastly increased, especially since the COVID-19 pandemic. However, this new teaching methodology has raised several issues that have not been resolved [8,9,10,11,12,13,14,15]. Numerous studies have investigated dental students’ attitudes towards online learning during the pandemic, and varied results have been reported. Furthermore, reports on the impact of online learning during the pandemic on students’ learning performance and comparing this with students’ performance before the pandemic are scarce [16]. The success of e-learning depends on many factors, which include the attitudes and interactive teaching styles of the faculty, as well as on the experience and attitudes of students with regards to technology [17]. A previous study indicated that online learning achieved better learning outcomes and was more highly satisfying than the in-person learning methods [18], while some other studies yielded no differences [19]. In a surveyed conducted by Merinchos et al. (2020) on participants of an online congress, they concluded that online learning was a good alternative to the traditional on-site learning, in improving the practical abilities as a dentist [20]. From a report by the U.S. Department of Education, a comparative study was conducted between examinations of online and in-person learning from 1996 to 2008 of the same course, they concluded that online learning could produce better or equivalent learning outcomes [16].

Since the implementation of online learning in higher education institutions in Malaysia is a new experience, few studies have been done to assess the students’ perspective regarding this learning method and its impact on students’ life. Moreover, no study has been done in Malaysia to compare students’ academic performances before and after online learning implementation. Therefore, the aim of this study was to assess IIUM dental students’ perspectives towards the implementation of online learning during COVID-19 and to evaluate the impact of online learning on students’ academic performance, by comparing their academic performance before and after the implementation of online learning. 

## 2. Materials and Methods

### 2.1. Ethical Considerations

Ethical approval was obtained from the IIUM Research Ethics Committee in January 2021 (Ref. No: IREC 2021-029). Informed consent was obtained from the participants through distributed questionnaires. Students’ matric numbers were code-protected by the principal investigator before the data was released to co-researchers. Thus, the questionnaire answers and examination results of the students were kept anonymous. 

### 2.2. Study Designs 

#### 2.2.1. Students’ Perspectives

All Year 1 until Year 5 IIUM dental students (292 students) were invited to answer the questionnaire. Online questionnaires were distributed through a WhatsApp group among IIUM dental students from academic session 2020/2021. The questionnaire was open from 6 January until 14 January 2021. The online questionnaire was created using a Google Form based on Schlenz et al. (2020) [14] and Khalil et al. (2020) [20], with some modifications. This questionnaire was divided into 6 parts. In Part 1, respondents were asked to provide their gender, year of study, and current location, whether on campus or in their hometown. Part 2 contained eight preliminary questions regarding online learning preferences. Part 3–6 contained 20 evaluative statements regarding management (students’ management of online learning), didactic benefits (the way students find online learning is helpful), motivations (students’ level of enthusiasm during online learning), and challenges (difficulties faced by the students during online learning). In response to each statement, a 5-point Likert scale was used, ranging between 1 (strongly disagree) and 5 (strongly agree).

#### 2.2.2. Students’ Academic Performance 

The impact of online learning was assessed by comparing examination results of Year 1, 2, and 3 dental students during face-to-face teaching (Professional Exam 2018/2019) and after online learning implementation (Professional Exam 2019/2020). A total of 231 students’ grade and examination result status for their professional exam were assessed across all subjects. Note that the students from the face-to-face cohort and online learning cohort were not the same person, but they took the same subjects for that corresponding year. Some subjects offered for Year 1 were not offered in Year 2 and Year 3. Therefore, this research only compared the students’ examination results from the same year. For example, academic performances of Year 1 2018/2019 students were compared with Year 1 2019/2020. 

The examination format during in-person learning and online learning sessions were the same and consisted of multiple-choice questions, short notes, and modified essay questions. Even though the examination questions were not the same, the topics, level of difficulty, and members of the faculty were the same. 

### 2.3. Statistical Analysis

Collected data were analyzed using IBM SPSS Statistics for Windows, Version 25.0. (IBM Corp., Armonk, NY, USA). For online questionnaires, the results from sociodemographic background (Table 1) and online learning preferences (Table 2) were analyzed into frequency and percentage. Descriptive statistics (frequency, percentage, mean and standard deviation) were used to analyze the results of each domain. The mean score for each domain was compared according to the sociodemographic background of the students using a Mann-Whitney U test and independent-samples *t*-test for comparison of the two groups, while a Kruskal-Wallis test and one-way ANOVA test were used for comparison of three or more groups. 

To analyze data from students’ academic performance, an independent *t*-test was used to compare means between exam grades of the previous cohort who received in-person learning during 2018/2019 with the cohort that underwent online learning during 2019/2020. The frequency of students with distinction/pass/fail and grade A/B/C/D/E/F were tabulated. The significance level was set at 0.05, with 95% confidence intervals.

## 3. Results

### 3.1. Sociodemographic Background and Students’ Perspectives Regarding Online Learning during COVID-19 

**Figure 1:**
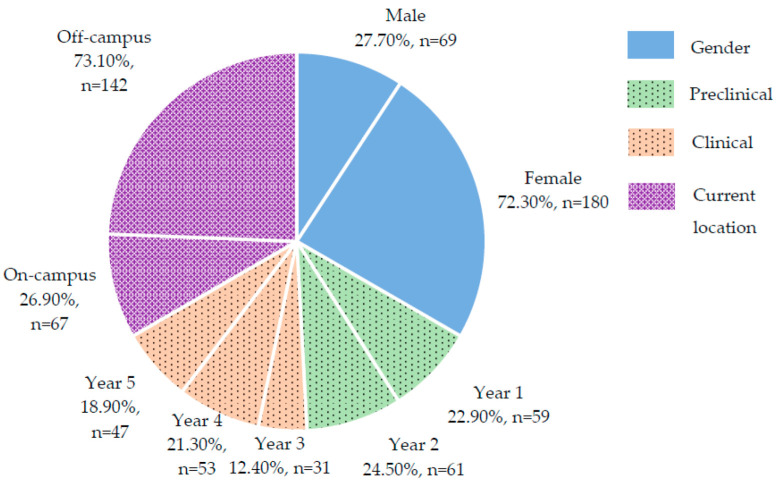
Sociodemographic background.

Figure 1 shows sociodemographic background of the respondents. A total of 249 Kulliyyah of Dentistry (KOD), IIUM students (69 males, 180 females) answered the online questionnaire, representing a response rate of 85.3%. From the respondents, 47.4% (n = 118) were preclinical students consisting of Year 1 and 2 students and 52.6% (n = 131) were clinical students consisting of Year 3, 4, and 5 students. 

**Figure 2:**
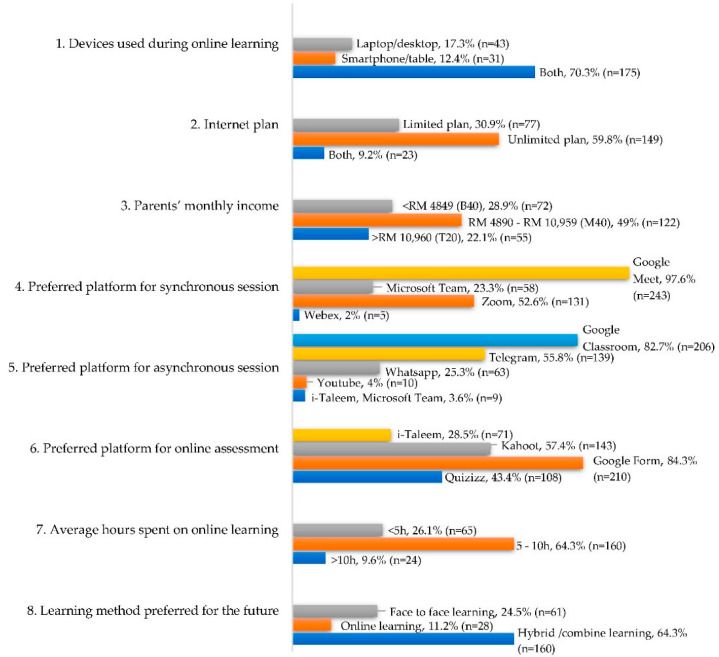
Online learning preferences.

Based on Figure 2, the majority of KOD students answering the questionnaire used both laptop/desktop computers and smartphones/tablets for their online learning and preferred subscribing to unlimited internet plans. Almost half (49%, n = 122) of the students belonged to the M40 socioeconomic group (parents’ monthly income that ranged from RM 4890 to RM 10,959). For synchronous learning sessions, students preferred using Google Meet, followed by Zoom. Meanwhile, for asynchronous sessions, students preferred pre-recorded learning sessions to be shared via Google Classroom, followed by Telegram and WhatsApp. Most students preferred their assessments to be conducted via Google form. More than half of the students spent an average of 5–10 h per day in online classes and self-study. Finally, 64.3% (n = 160) of the students preferred hybrid learning methods instead of online learning alone after the pandemic.

### 3.2. Students’ Perspectives on Management of Online Learning, Didactic Benefits, Motivations, and Challenges 

#### 3.2.1. Domain 1-Management of Online Learning

Table 1 shows students’ perspectives regarding online learning during COVID-19. Most of the students (62.7%, n = 156) agreed that trial runs conducted by lecturers in the first two weeks before starting the full e-learning sessions prepared them well for the online class. More than half of the students agreed that they were able to prepare themselves well for online classes and were able to follow the content well during class. About half of the respondents were neutral, and another half agreed that the image and sound quality of online learning was good. Most students (77.1%, n = 192) agreed that basic computer skills were necessary for smooth online learning.

#### 3.2.2. Domain 2-Didactic Benefits

The majority of students found that online learning was effective for learning theoretical subjects but did not feel that online learning alone was able to prepare them well for practical sessions or treating patients. Some students felt more confident in asking questions during online class. Furthermore, 63.9% (n = 159) of the students strongly agreed that lecture recordings were beneficial for self-study, while 81.2% (n = 202) of the students agreed that remote teaching tools such as Kahoot were beneficial.

#### 3.2.3. Domain 3-Motivations 

Less than half of the students found that online learning motivated them to study. Most of the students (44.6%, n = 111) were undecided if they were excited or not for online learning. Only 33.8% (n = 84) of students agreed that being at home with family motivated them to learn. Most students (53%, n = 132) agreed that the lack of body language cues between the lecturers and students demotivated them from focusing during online class.

#### 3.2.4. Domain 4-Challenges

A total of 42.1% (n = 105) of KOD students had problems with their internet connection during online learning, while 29.3% (n = 73) did not have problems with their internet connection. Most of the students (57.4%, n = 143) did not suffer an extra financial burden from the implementation of online learning and also agreed that they had difficulty concentrating during long periods of online learning, while increased screen time caused eye fatigue. Furthermore, most students felt that home was an unconducive environment for studying, as they were easily distracted during class. Online learning also limited students’ social interaction with classmates and lecturers compared to face-to-face classes.

Table 2 shows cross tabulation data between management of online learning, didactic benefits, motivations, and challenges scores according to sociodemographic background (gender; year of study; current location) of the students. Females scored higher than males in the management of online learning, didactic benefits, and motivations, while males scored higher for the challenges domain compared to females. However, only the management of online learning domain showed a significant difference (*p*-value = 0.014) with the gender of the respondents. Year 1, year 2, and year 3 scored the highest for management of online learning and didactic benefits, domain challenges, and motivations, respectively. Corresponding to the year of study, significant differences (*p*-value < 0.05) were found in the management of online learning and motivations. Off-campus students had higher mean scores for domain challenges compared to on-campus students. For other domains, on-campus students had higher mean scores for domain 1, 2, and 3, while off-campus had higher mean for domain 4. Significant differences were shown in didactic benefits (*p*-value = 0.005) and motivations (*p*-value = 0.019) scores when comparing the on-campus and off-campus students.

### 3.3. Comparison of Students’ Academic Performance: Online Learning Cohort Versus Face-to-Face Cohort 

The results of the professional exam 2018/2019 taken during face-to-face session were compared with the results from the professional exam 2019/2020 taken after the implementation of online learning (Table 3). Among the four subjects taken by Year 1 students, only oral biology showed significant improvement in examination results between year 2018/2019 (face-to-face learning) and 2019/2020 (online learning) (*p*-value ≤ 0.001). In year 2, significant differences were observed in pro-exam results between face-to-face learning compared to online learning in several subjects (*p*-value < 0.05). The mean score for microbiology and pharmacology increased significantly, but the dental material score decreased significantly during online learning. Year 3 GMGS results of the online learning cohort showed a significantly decline in the mean score compared to face-to-face students. 

More students received distinctions after the implementation of online learning in four subjects (Table 4) compared to face-to-face learning. The number of students who failed microbiology and pharmacology dropped to zero after the implementation of online learning. 

## 4. Discussion 

### 4.1. Students’ Perspectives Regarding Online Learning

Even though little evidence has been reported regarding the impact of the pandemic on higher education, one study has shown a possible positive effect of the COVID-19 confinement on students’ performance [21]. Growing evidence claims that e-learning is as effective as traditional methods [14]. A review investigating the barriers and enablers of e-learning concluded that distant education might enhance learning and performance due to its flexibility and accessibility [22]. Through online learning, students’ can improve their knowledge and enhance their ability in applying the knowledge that they obtain during the online teaching session in a clinical setting [20].

For the successful implementation of online learning, basic computer and typing skills are necessary [23,24,25]. In this study, most students found that basic computer skills and trial runs conducted before the start of e-learning sessions to familiarize them with the online system were helpful for online learning, as they had time to prepare themselves with study materials before the start of class. Well-designed online learning provides students with more time to access the study material beforehand [25]. A good quality internet service is necessary to utilize online content and prevent technical problems arising from a slow internet connection [21]. In this study, although nearly half of the students had internet connection issues, it did not affect the learning process much; the majority of students had good video and audio quality during online learning and followed the lectures well. 

Online lectures are useful for theoretical learning, but the absence of clinical practice and lab sessions hinders the mastery of practical concepts [21], and online learning was less appealing to students due to its limitations with respect to the practical aspects of learning in the lab/clinical environment. In this study, many students, especially clinical students, agreed that online learning was a good option for theoretical study, but that this alone did not provide students with the confidence to treat patients. Ease of access and use of online tools are important for online learning [26]. For KOD students, online teaching tools such as Kahoot are commonly used for quizzes, but most students prefer Google Form as a medium for online assessment, due to the unlimited response time compared to Kahoot. Meanwhile, students preferred Google Meet for live session classes, due to its convenient features and unlimited time sessions. Moreover, recorded lectures, seminars, and presentations helped students during their self-study sessions.

Self-regulatory behaviors and e-learning motivation are factors that strengthen the preference for online learning [27]. In this study, most students were unsure whether they became more motivated and excited to learn using online mediums; however, they agreed that they became more confident to ask questions during online classes. Some students preferred typing questions in the chat box during live online classes, rather than speaking in class [16]. Students were satisfied with online learning, as they were able to study at their comfort and spend more time with their family [19]. However, the percentage of KOD students that agreed or disagreed that being at home motivated them to study was nearly the same.

The online learning process poses some challenges to students. Too much screen time is a pitfall, as the blue light emitted from electronic devices may cause eyestrain for some people [28]. Long periods of online learning reduced students’ focus and affected their eye health. Moreover, for students living off-campus, the surrounding home environment home was a distraction during online classes. Some students preferred active interaction with lecturers during face-to-face learning [21]. Most students agreed that online learning limited their social interactions, and a lack of body language cues such as eye contact with the lecturers demotivated them during class. This lack of interaction made students feel isolated; therefore, the faculty should promote various interactions using the interactive features of online learning platforms [16]. Some students experienced an extra financial burden and limited internet quota with online learning [25]. However, the majority did not suffer these problems, as most belonged to the M40 socioeconomic group and used unlimited internet plans for online learning.

Through this study, we can understand the students’ acceptance of the changes in their study methods and, based on that, some improvement can be made to the online learning system. However, this questionnaire consists only of structured questions regarding online learning, and students may have different perspectives that were not included in the questionnaire. In future study, students’ subjective opinions regarding online learning may need to be assessed, as this could affect the outcome of the study.

### 4.2. Students’ Academic Performance during the Pandemic

The worldwide COVID-19 outbreak immediately affected dental students’ education, forcing nearly all related universities to postpone their clinical and practical sessions and continue the learning process through an online approach. Limited evidence is available on the effect of the pandemic on higher education; however, Gonzalez et al. (2020) [22] and Regmi et al. (2020) [23] reported that COVID-19 enhanced student academic learning and performance. The ease of access and flexible learning pattern made students find online learning fulfilling [29]. According to Pei and Wu (2019) [30], online learning was superior to conventional face-to-face learning, purely based on knowledge and skill outcomes, specifically in medical education. 

In this study, students’ academic performance showed a significant improvement overall after the implementation of online learning. In Year 1, online learning increased student performance in oral biology significantly. Oral biology (OB) is a science-related subject that develops a mechanistic understanding of oral tissue development and function in relation to tissue structure, oral health, and disease [31]. Despite this subject requiring practical learning, information can be successfully conveyed to the students through online platforms, based on their increased performance with online learning. Zheng et al. (2021) also reported that students were equally or more likely to get an A online compared to face-to-face learning in the subject “anatomy and histology”, which had the least student support for an online format [16].

In Year 2 students, microbiology and pharmacology showed significant improvements. These subjects require theoretical instead of practical learning. A recent study showed similar results, where clinical microbiology e-learning was well received and had a positive impact on examination performance [32]. A slight decrease in students’ performance was observed in the dental material subject with online learning. Dental materials is one of the most difficult subjects, due to its technical nature and the need for a high comprehension ability [33]. This might explain the poor performance in this subject when taken online. 

GMGS subjects require practical and physical learning, in addition to theoretical learning. Implementation of virtual learning for GMGS is challenging, hence students’ performances were slightly affected with online learning compared to face-to-face learning. Anatomy is a visual and three-dimensional subject; the small smartphone screen size may hinder proper understanding of the subject [34]; possibly missing the real objectives of the subject. A lack of history taking and physical examinations of patients due to MCO affected student performance in the objective structured clinical examination (OSCE) subject. Although some studies reported that both students and professors appreciated virtual learning approaches, remote teaching cannot replace clinical training [35]. It is clear, based on this report, that pre-clinical year students (Year 1 and Year 2) did well in examinations during the online learning period compared to clinical year students (Year 3). However, the data for students’ academic performance was compared in a one-year period only. Thus, it is inconclusive to state that online learning influences students’ academic performance. A longer period of study is required to re-evaluate the students’ academic performance for a better and more precise outcome. 

The majority of publications on dental education online learning during the COVID-19 pandemic have focused on the learning satisfaction of the students, or their lecturers, whilst forgetting to assess scientifically its implications for academic performance [16]. Much of the available literature is based on self-reported data from students. Overall, students and lecturers agreed that student learning had worsened at the Harvard University dental faculty [17]; this sentiment was repeated in Pakistani dental and medical faculties [16]. While both studies are important, they account mostly for students’ perceived learning gain, which can be inaccurate compared to actual learning gain data [16], which leaves a gap for assessing the actual effects of online learning on performance outcomes [16]. The present study examined students’ perspectives towards online learning and their academic performance in general. Even though students faced some difficulties and challenges, they also showed a positive acceptance towards online learning. In relation to the students’ academic performance, pre-clinical year students showed an increment in their academic performance, while this was contradicted in clinical year students. Moreover, some subjects in both pre-clinical and clinical years did not show any significant difference in students’ performance with online learning and in-person learning. In accordance with the hypothesis, online learning did influence IIUM dental students’ academic performance: a significant increase in good grades and the frequency of distinctions and passes was observed in pre-clinical year students. Other benefits and drawbacks of online learning need to be studied further, to determine students’ academic performance and assess the effectiveness of online education systems.

This was a relevant study for assessing students’ performance after major changes had occurred to learning methods, due to the pandemic. However, the data were compared in a one-year period only. In order to achieve a better result regarding the impact of online learning on students’ academic performance, this type of study should be done in a longer period of time. Moreover, this study only assessed IIUM dental students, which may not reflect outcomes from other schools and disciplines; thus, studies with larger study populations may be needed in the future.

## 5. Conclusions

With the pandemic persisting for almost two years, education, specifically dental education, had to be continued via online means. Despite the many challenges encountered by students with this change, dental students generally had a positive response towards online learning. Academic performance, as a measure of comparison of before and after online learning, improved in some subjects, remained the same in some, and declined in others. Nevertheless, other factors influencing academic performance should be assessed in future studies. 

## Figures and Tables

**Table 1:** Assessment of Domain 1 (management of online learning), Domain 2 (didactic benefits), Domain 3 (motivations), and Domain 4 (challenges).

Statements	1 n (%)	2 n (%)	3 n (%)	4 n (%)	5 n (%)	Mean ± SD
Domain 1—Management of online learning						
1. Trial run of online learning during the first two weeks prepared me well for the online class.	3 (1.2)	19 (7.6)	71 (28.5)	96 (38.6)	60 (24.1)	3.77 ± 0.943
2. I was able to prepare myself well in advance for online learning.	1 (0.4)	26 (10.4)	65 (26.1)	113 (45.5)	44 (17.7)	3.69 ± 0.895
3. I can follow the online teaching content well.	8 (3.2)	35 (14.1)	80 (32.1)	93 (37.7)	33 (13.3)	3.43 ± 0.994
4. The image and sound quality of online learning was good.	7 (2.8)	29 (11.6)	103 (41.4)	77 (30.9)	33 (13.3)	3.40 ± 0.954
5. Computer skills are necessary for smooth online learning.	5 (2.0)	6 (2.4)	46 (18.5)	91 (36.5)	101 (40.6)	4.11± 0.926
Domain 2—Didactic Benefits						
1. Online learning is a good option for learning theoretical subjects.	8 (3.2)	21 (8.4)	68 (27.3)	103 (41.4)	49 (19.7)	3.66 ± 0.992
2. By participating in online learning alone, I feel well prepared for the practical session.	43 (17.3)	80 (32.1)	92 (36.9)	25 (10.0)	9 (3.6)	2.51± 1.009
3. Online learning makes me braver to ask questions in class.	9 (3.6)	29 (11.6)	80 (32.1)	75 (30.1)	56 (22.5)	3.56 ± 1.073
4. Lecture recordings during online classes are beneficial for self-study.	2 (0.8)	1 (0.4)	23 (9.2)	64 (25.7)	159 (63.9)	4.51 ± 0.746
5. Online teaching tools (e.g., Kahoot, EdPuzzle, TED-Ed) are beneficial.	2 (0.8)	3 (1.2)	42 (16.9)	105 (42.2)	97 (39.0)	4.17 ± 0.807
Domain 3—Motivations						
1. Online learning motivates me to learn.	22 (8.8)	35 (14.1)	91 (36.5)	66 (26.5)	35 (14.1)	3.23 ± 1.129
2. I feel more excited to learn every day using online learning.	20 (8.0)	46 (18.5)	111 (44.6)	45 (18.1)	27 (10.8)	3.05 ± 1.059
3. Being at home with family motivates me to learn better during online learning.	23 (9.2)	45 (18.1)	97 (39.0)	41 (16.5)	43 (17.3)	3.14 ± 1.179
4. Lack of body language (e.g.,: face expression, hand gesture, eye contact) demotivates me during online classes.	10 (4.0)	31 (12.4)	76 (30.5)	84 (33.7)	48 (19.3)	3.52 ± 1.063
Domain 4—Challenges						
1. I face problems during online learning due to my internet connection.	22 (8.8)	51 (20.5)	71 (28.5)	75 (30.1)	30 (12.0)	3.16 ± 1.149
2. I suffer extra financial burdens due to online learning.	58 (23.3)	85 (34.1)	61 (24.5)	22 (8.8)	23 (9.2)	2.47 ± 1.205
3. I have difficulty concentrating during long periods of online learning.	3 (1.2)	13 (5.2)	32 (12.9)	85 (34.1)	23 (9.2)	4.20 ± 0.936
4. The surrounding environment distracts me during online class.	14 (5.6)	25 (10.0)	70 (28.1)	79 (31.7)	61 (24.5)	3.59 ± 1.129
5. Increased screen time during online learning worsens my eye condition.	7 (2.8)	24 (9.6)	67 (26.9)	78 (31.3)	73 (29.3)	3.75 ± 1.068
6. Online learning limits my social interaction with the teacher/classmates in the class.	15 (6.0)	13 (5.2)	60 (24.1)	77 (30.9)	84 (33.7)	3.81 ± 1.140

SD = standard deviation.

**Table 2:** Management of online learning, didactic benefits, motivations, and challenges scores according to the sociodemographic background of the students.

Socio Demo-Graphic Characteristics	N	Domain 1 Management of Online Learning Score Mean (SD)	*p*-Value	Domain 2 Didactic Benefits Score Mean (SD)	*p*-Value	Domain 3 Motivations Score Mean (SD)	*p*-Value	Domain 4 Challenges Score Mean (SD)	*p*-Value
Gender			0.014 *		0.088		0.091		0.207
Male	69	17.65 (3.07)		17.93 (2.75)		12.52 (2.45)		21.43 (4.05)	
Female	180	18.70 (3.38)		18.60 (2.97)		13.11 (2.82)		20.80 (4.21)	
Year of study			0.001 *		0.146		0.008 *		0.061
Year 1	57	19.52 (3.11)		20.93 (2.82)		13.14 (2.74)		20.93 (4.40)	
Year 2	61	16.87 (3.63)		17.79 (2.98)		11.75 (3.03)		22.28 (3.58)	
Year 3	31	18.58 (3.63)		19.03 (3.17)		13.87 (2.63)		20.13 (5.03)	
Year 4	53	18.87 (3.00)		18.94 (2.69)		13.00 (2.12)		20.53 (3.84)	
Year 5	47	18.40 (2.70)		18.77 (2.93)		13.57 (2.46)		20.40 (4.12)	
Current location			0.928		0.005 *		0.019 *		0.299
On-campus	67	18.55 (3.32)		19.09 (3.33)		13.70 (2.48)		20.54 (4.58)	
Off-campus	182	18.36 (3.33)		18.16 (2.72)		12.66 (2.77)		21.14 (4.01)	

* p < 0.05, SD = standard deviation.

**Table 3:** Professional exam results.

			Examination Scores (Maximum 100)			
**Year 1 Professional exam results**						
**Exam**	**N**		**Anatomy**	**Physiology**	**Biochemistry**	**Oral** **Biology**
Face-to-face (Pro Exam 2018/2019)	60	Mean (SD)	59.07 (12.73)	62.65 (11.24)	60.32 (9.41)	63.77 (9.12)
Online learning (Pro Exam 2019/2020)	62	Mean (SD)	62.03 (11.07)	61.43 (8.48)	61.92 (11.66)	71.25 (8.49)
		*p*-value	0.172	0.502	0.406	0.000 *
**Year 2 Professional exam results**						
**Exam**	**N**		**Dental** **Material**	**Pathology**	**Microbiology**	**Pharmacology**
Face-to-face (Pro Exam 2018/2019)	55	Mean (SD)	65.48 (5.84)	72.05 (9.88)	65.58 (8.41)	67.20 (7.93)
Online Learning (Pro Exam 2019/2020)	56	Mean (SD)	62.88 (6.98)	71.93 (9.30)	72.23 (7.78)	74.45 (7.94)
		*p*-value	0.036 *	0.945	0.000 *	0.000 *
**Year 3 Professional exam results**						
**Exam**	**N**		**General Medicine General Surgery**			
Face-to-face (Pro Exam 2018/2019)	59	Mean (SD)	72.54 (4.45)			
Online Learning (Pro Exam 2019/2020)	55	Mean (SD)	67.98 (4.68)			
		*p*-value	0.000 *			

SD = standard deviation, * *p*-value < 0.05.

**Table 4:** Examination results for selected Year 1, 2, and 3 subjects, with a significant mean score improvement with online learning.

	Oral Biology (Year 1)		Microbiology (Year 2)		Pharmacology (Year 2)		General Medicine and General Surgery (Year 3)	
	Face-to- Face, N	Online Learning, N	Face-to- Face, N	Online Learning, N	Face-to- Face, N	Online Learning, N	Face-to- Face, N	Online Learning, N
Distinction	2	6	2	9	2	12	3	0
Pass	55	56	52	47	52	44	56	55
Fail	3	0	1	0	1	0	0	0
AP	0	0	0	0	0	0	0	0
Total	60	62	55	56	55	56	59	55

AP refers to absence with permission.

## Data Availability

Data of the study will be shared upon request to the corresponding author.
